# Case report: Development of clonal hematologic disorders from inherited bone marrow failure

**DOI:** 10.3389/fonc.2024.1420666

**Published:** 2024-09-09

**Authors:** Jaroslav Cermak

**Affiliations:** Department of Clinical Hematology, Institute of Hematology and Blood Transfusion, Prague, Czechia

**Keywords:** inherited bone marrow failure, cytopenia, diagnosis, treatment, myelodysplastic syndromes, transplantation

## Abstract

**Introduction:**

Inherited bone marrow failure (IBMF) syndromes are caused by mutations forming pathologic germline variants resulting in the production of defective hematopoietic stem cells (HSC) and in congenital failure in the production of one or more blood lineages. An acquisition of subsequent somatic mutations is determining further course of the disease. Nevertheless, a certain number of patients with IBMF may escape correct diagnosis in childhood, especially those with mild cytopenia and minimal clinical features without non-hematologic symptoms. These patients usually present in the third decade of life with unexplained cytopenia or myelodysplastic syndrome (MDS).

**Methods and results:**

We report 2 patients with IBMF who were correctly diagnosed between 20 and 40 years of age when they were referred with progressive MDS with adverse prognostic factors that affected their outcome.

**Discussion:**

IBMF syndromes should be excluded in all patients below 40 years of age with unexplained cytopenia. Early hematopoietic stem cell transplantation (HSCT) is the treatment of choice in these patients.

## Introduction

Inherited bone marrow failure (IBMF) syndromes are caused by mutations forming pathological germline variants. The result is a production of defective hematopoietic stem cells (HSCs) and stressed hematopoiesis (1). The pathogenesis of HSC progenitor cells failure depends on the type of the lesion caused by patient’s specific germline variant. The lesions may result in defective DNA cross-link mutation repair [Fanconi anemia (FA)], defective telomere maintenance [dyskeratosis congenita (DC)], ribosomopathies [Shwachman Diamond syndrome (SDS) and Diamond Blackfan anemia (DBA)], defect of terminal maturation (congenital neutropenia), defects of signaling receptors, cell growth regulation, and cell transcription (2). A germline genetic defect is responsible for an activation of DNA damage response. Less severe and potentially repairable damage activates cell senescence and cell growth arrest via p53-mediated pathway (3). An extensive DNA damage results in excessive p53-induced pathway. Both decreased stem cell fitness and stem cell depletion lead to cytopenia and marrow failure (2). Subsequent acquisition of somatic mutations is crucial for the development of an MDS clone. These somatic alterations may have an adaptive outcome resulting from the correction of germline phenotype or maladaptive outcome connected with an increased risk of neoplastic clonal evolution (2). The risk of development of myelodysplastic syndromes (MDSs) or myeloproliferative neoplasms (MNs) is from 40- to 7,700-fold higher than in normal population (4). IBMF is commonly diagnosed during childhood. However, some patients with discrete symptoms may escape an early diagnosis and present as young adults with unexplained cytopenia or MDS. Here, we report two patients with IBMF who were correctly diagnosed between 20 years and 40 years of age when they were referred to our department with the diagnosis of MDS.

## Patients

### Case report 1

**Figure 1:**
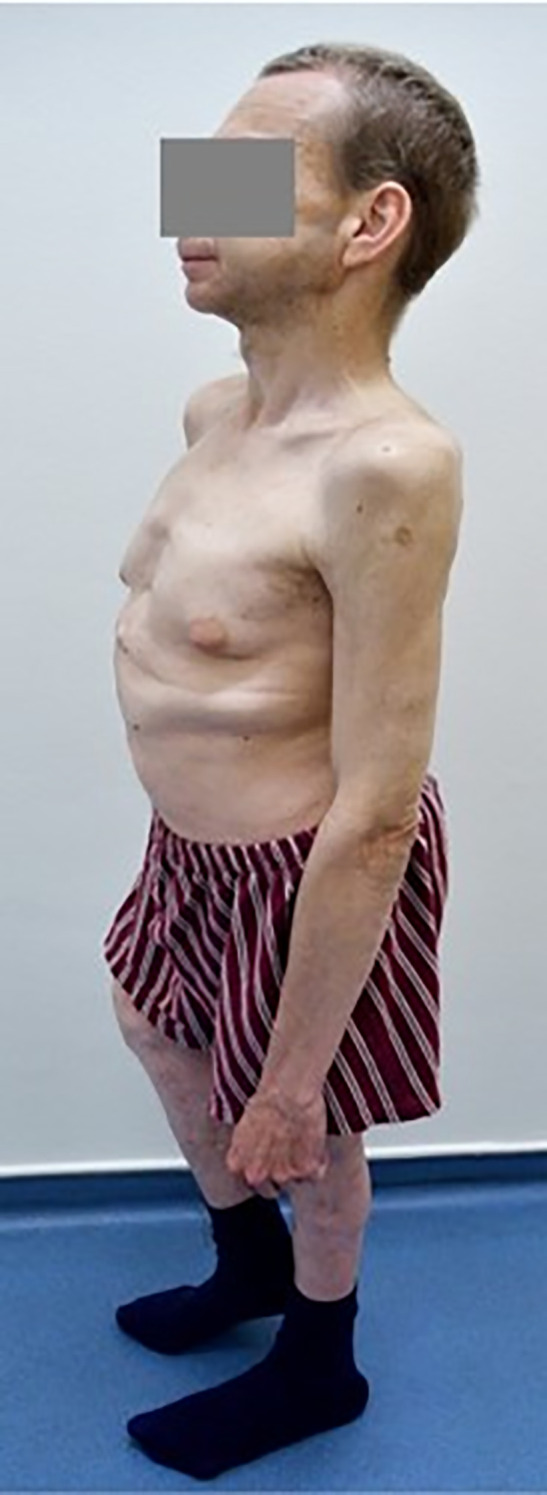
Short stature and malformations of the skelet in the patient with SDS.

The patient was followed at the regional department for sick children since the age of 4 years with mild cytopenia, growth retardation, repeated infections, and gut discomfort. The patient was consulted with Clinics for Pediatric Hematology, and SDS was hypothesized as one of the possible reasons of cytopenia. However, this suspicion was never confirmed, and a final diagnosis of MDS RCC (refractory childhood cytopenia) was established. At that time, his peripheral blood counts were as follows: Hb, 113 g/L; WBC, 2.66 × 10^9^/L; NS, 35%; PLT, 70 × 10^9^/L. Bone marrow was described as “slightly hypocellular without marked signs of dysplasia”. As an adult, the patient was followed at the regional hematology department. He suffered from recurrent pulmonary infections without substantial worsening of the cytopenia. He required the first red blood cell (RBC) transfusion at the age of 36. At that time, he was referred sent to the Institute of Hematology in Prague because of progressive cytopenia with dg. MDS RCMD (refractory cytopenia with multilineage dysplasia) and del(5q). The patient’s height was 146 cm with skeletal abnormalities of chest and spine (**Figure 1**). Peripheral blood counts and results of bone marrow investigation are summarized in **Table 1**. Investigation of SBDS gene revealed biallelic mutation within exon 2: c.183-184TA>CT. The diagnosis of MDS with excess of blasts II. (IB2) developed from SDS was established. The patient had multiple karyotype abnormalities and mutations of driver genes, including a biallelic mutation of TP53. His IPSS-R score was 8.5 (5).

**Table 1:** Peripheral blood and bone marrow findings in SDS patient (Hb, hemoglobin; RBC, red blood cells; RTC, reticulocytes; WBC, white blood cells; NS, neutrophil segments; PLT, platelets; NGS, next-generation sequencing).

Peripheral blood counts	Hb, 127 g/L; RBC, 4.17 × 10¹²/L; RTC, 0.83% (34.6 × 10^9^/L) WBC, 1.2 × 10^9^/L; diff.: NS, 13%; myeloblasts, 1% PLT, 36 × 10^9^/L
Bone marrow	Normocellular with trilineage dysplasia, 13% myeloblasts Cytogenetics—46,XY,del(5)(q13;q33) [8] 56,XY,+1,del(5)(q13;q33),+8,+9,+10,+i(11)(q10),+14,+15,+20,+21,+22[10] 46,XY [4] molecular genetics: SBDS gene− exon 2: c.183-184TA>CT NGS: DNMT3A 25%, TP53 biallelic 43%, ASXL1 11%

The patient underwent combination chemotherapy (Daunorubicin 90 mg day 1–3 + Cytarabine 150 mg day 1–7). Bone marrow aspiration performed on day (D) 21 of induction chemotherapy revealed hypoplastic bone marrow with 11% of blasts, and the patient underwent immediately allogeneic peripheral blood stem cell transplantation (PBSCT) from HLA-matched unrelated donor using conditioning regimen Fludarabine + Melphalan + antithymocyte globulin (ATG). He was engrafted on D 23 after SCT in morphological remission, but within 3 months, he had mixed chimerism again despite repeated donor lymphocyte infusions. Nonetheless, he was relatively quite well with skin and liver chronic graft versus host disease (GVHD) grade II until D 485 after PBSCT when he relapsed. Best supportive care only was indicated because of his poor performance status. The patient died D 510 after PBSCT from sepsis and multiple organ failure.

### Case report 2

At the age of 6 years, this patient underwent surgical removal of an abundant thumb on the right hand. Preoperative investigation of peripheral blood counts revealed macrocytosis (MCV=107 fl) and slightly decreased PLT count (91 × 10^9^/L). The patient was asymptomatic and was infrequently followed by a regional pediatrician. At the age of 17, the patient was referred to Clinics for Pediatric Hematology with pancytopenia and macrocytosis (Hb= 105 g/L, MCV = 107 fl, WBC = 2.4 × 10^9^/L, PLT = 71 × 10^9^/L). A chromosome fragility assay showed an increased rate of chromosomal breaks [5% spontaneous, 8% with diepoxybutane (DEB)]. The patient was referred to the Institute of Hematology with suspected FA, but she canceled a planned appointment and disappeared for 2 years. She returned at the age of 20 with fatigue, dyspnea, and recurrent infections. Peripheral blood counts and results of bone marrow investigation are summarized in **Table 2**. Investigation of FANCA gene revealed biallelic mutation c.3788-3490 (del TcT).

**Table 2:** Peripheral blood and bone marrow findings in FA patient (Hb, hemoglobin; RBC, red blood cells; RTC, reticulocytes; WBC, white blood cells; NS, neutrophil segments; PLT, platelets; NGS, next generation sequencing).

Peripheral blood counts	Hb, 101 g/L; RBC, 3.69 × 10¹²/L; RTC, 0.35% (12.9 × 10^9^/L); MCV, 89 fl WBC, 1.5 × 10^9^/L; diff.: NS, 27%; myeloblasts, 7% PLT, 91 × 10^9^/L
Bone marrow	Normocellular with increased percentage of erythropoiesis, decreased rate of megakaryopoiesis, trilineage dysplasia, 8% myeloblasts cytogenetics: 46,XX,der(11)t(1;11)(q31;q23)—MLL, del(13)(q12q21 [22] FANCA gene: biallelic mutation c.3788-3490 NGS: RUNX1 pThr11Asn—45%

The diagnosis of MDS with excess of blasts II. (IB2) progressing from FA was established. The patient had deleted MLL gene and RUNX1 gene mutation; her IPSS-R score was 5 (5). She underwent upfront allogeneic PBSCT from HLA-matched related donor with reduced conditioning (Fludarabine + Cyclophosphamide + ATG). Engraftment was achieved on D 35 after SCT. The patient did not have any complications after SCT except mild acute GVHD in gut. She is currently 8 years after SCT and doing well.

## Discussion

An acquisition of somatic mutations determines the subsequent course of the disease in IBMT patients. Correction of the germline defect can be mediated via gene reversion or back mutation, deletion of point mutation, or by second site mutation suppressing the effect of the original mutation (1, 6). Compensation of the germline defect indirectly by somatic alteration of other genes reduces impact of the germline defect, e.g., acquisition of i[7q] and del20q in SDS (7). On the other hand, somatic alteration directly in p53 gene or in a gene silencing p53 effectivity may allow increased cell senescence. For example, chromosome 1 gain with trisomy of MDM4 gene downregulates p53 signaling pathway (7). Clonal somatic changes may also increase cell vulnerability to acquire further genetic alterations, especially mutations of the so-called driver genes, which play an important role in the regulation of proliferation, differentiation, and cell apoptosis (8, 9). The most frequently acquired cytogenetic clones are 20q−, i[7q] in SDS, and 1q+, 3q+, and −7/7q− in FA. Complex cytogenetic aberrations, monosomy 7, and 3q+ represent high-risk changes. Most common somatic gene mutations are TP53, EIF6, RUNX1, UZAF1, BCOR, STAG2, ASXL1, NRAS/KRAS, and SETBP1 mutations. Biallelic TP53 alterations, RUNX1, SETB1, ASXL1, and U2AF1 gene mutations are now considered to be high-risk mutations. Progression towards MDS/AML has been described in 30%–40% patients aged 30–40 years with SDS, FA, or GATA-2 mutations (10). SCT is the only curative treatment, and 10 years survival is approximately 75% according to the EBMT study (11). In a retrospective study of transplanted patients below 40 years of age, IBMF preceded development of MDS in 4%–7% of patients with SDS (12) and in 10%–15% patients with aplastic anemia (10).

Shwachman–Diamond syndrome (SDS) is an inherited ribosomopathy characterized by bone marrow failure, exocrine pancreatic deficiency, and skeletal dysplasia (osteopenia, short flared ribs, delayed ossification, and low height). In 90% of patients with SDS, a biallelic mutation of the SBDS gene leads to impaired joining of 60S and 40S ribosome subunits. This results in impaired translational capacity and upregulation of TP53 activity and senescence and apoptosis of hematopoietic stem cells (HSCs). Subsequent recurrent mutations of the EIF6 gene may alleviate the blocking effect of eIF6 on subunits joining and correct to some extent the germline defect similarly as does the acquisition of i[7q] and del20q (13). On the other hand, patients with SDS have an increased risk of developing myeloid malignancies and clonal hematopoiesis caused by mutations bypassing or alleviating intrinsic fitness caused by SBDS mutation. TP53 mutations are generally missense, and TP53 inactivation is associated with increased leukemogenic potential (14). Isochromosome 7q and del20q are the most recurrent cytogenetic abnormalities, where i[7q] results in duplication of SBDS gene. However, an adverse prognosis is connected with monosomy 7 and del(7q) (10). In the French SDS Registry (15), the incidence of myeloid malignancy was 24.3% and 36% at the age of 20 years and 30 years, respectively. In patients who developed leukemic progression, the prognosis is poor, with an analysis of the North American Registry reporting median survival of 7.7 years and 1 year for MDS and AML, respectively (16). Allogeneic hematopoietic stem cell transplantation (HSCT) is the only potential curative therapy for IBMF. It has been shown that the results of transplantation performed prior to development of MDS were significantly better than those performed during leukemic progression (5-year overall survival of 70.7% vs. 28.8%, respectively) (13, 16). There was no difference in results between upfront HSCT and HSCT after cytoreductive chemotherapy. The experience with bridging therapy using hypomethylating agents (HMAs) before SCT is limited (13).

The problem in the case of our patient was an insufficient diagnostic workup in childhood and the insistence on diagnosis of MDS with 5q deletion, whereby no further follow-up karyotypes and NGS were performed. Del5q has rarely been described in SDS patients; however, no systematic functional analyses have confirmed a causal link between haplo-insufficiency of a single gene and development of clonal advantage as in 5q− syndrome (17). The presence of a high load of biallelic TP53 mutation contributes to a poor prognosis even in transplanted SDS patients as seen together with multiple karyotype abnormalities in our patient. Mutations of DNMT3A and ASXL1 driver genes have also been described in several SDS patients (6.7% of patients in the study by Kennedy et al.) (7).

Fanconi anemia (FA) is an IBMF with DNA instability caused by biallelic mutations in one of 22 FANC pathway genes that are involved in DNA damage repair and stress response (18). Activation of FANC pathway forms “FA core” multiprotein complex that activates D2/I heterodimer, which interacts with downstream proteins including BRCA2. The result is a replication-coupled excision of the damaged locus followed by homologous recombination. FA patients have usually normal blood counts at birth, and the first signs of bone marrow failure (macrocytic anemia and thrombocytopenia) occurs within the first decade of life (median age of onset is 7 years). Approximately 80% of FA patients will develop bone marrow failure by the age of 20. Skeletal malformations include abnormal thumbs, absent radii, short stature, skin hyperpigmentation, and abnormal facial features. Approximately 30% of FA patients do not have any of the classic physical findings, but the diepoxybutane (DEB) chromosome fragility assay showing increased chromosomal breaks could be diagnostic (19). One-half of the FA patients may develop gain of chromosome 1q driving enforced MDM4 oncogene expression that leads to repression of p53 response. p53 attenuation confers an advantage to the 1q+ clone that may expand, repopulate exhausted bone marrow, and form a base for acquisition of additional driver events: RUNX1 inactivation, 3q+/EVI1 mutation, 7q deletion, and finally, development of leukemic progression. Patients with FA have increased risk of hematologic malignancies, head and neck squamous cell carcinoma, gynecological cancers, and other solid tumors. The estimated incidence of AML in FA patients is 15%–20% in 40 years, and the cumulative incidence of MDS and solid tumors is 40% and 16% in 50 years, respectively (20). HSCT is also a potential curative treatment in FA patients, but the indication must be considered carefully. Combination chemotherapy and radiation including conditioning regimens are associated with excessive toxicity and an increased rate of solid tumors after HSCT. An optimal time for indication of HSCT is progressive cytopenia before development of adverse cytogenetic aberrations and gene mutations. The 3-year survival of FA patients transplanted following reduced conditioning from HLA-matched related donor is more than 90% (20). The problems in the history of the disease in our FA patient were insufficient diagnosis with the lack of NGS investigation and poor cooperation of the patient. Progressive cytopenia, increased percentage of blasts, and the presence of the RUNX1 gene mutation were adverse factors that forced us to indicate an immediate HSCT in the patient. On the other hand, the availability of HLA-matched related donor and only mild excess of bone marrow (BM) blasts enabled us to use reduced conditioning and avoid toxic cytoreductive treatment. These factors clearly played an important role in the outcome of transplantation.

## Conclusions

IBMF syndromes are characterized by congenital failure in the production of one or more blood lineages. The clinical manifestations of the IBMF may vary according to the type and number of blood cell lines involved. In some IBMF syndromes, systemic non-hematologic manifestations, including congenital malformations and impaired development, may be present. Despite these features, a certain number of patients with IBMF may escape correct diagnosis, especially those with mild cytopenia and minimal clinical features without non-hematologic symptoms. The need for regular follow-up of these patients is not usually stressed sufficiently. IBMF syndromes represent a qualitatively impaired functional state and a basis for somatic alterations. This process can promote clonal hematopoiesis by improving the competitive fitness of specific hematopoietic stem cell clones. All children with unexplained anemia or cytopenia should by investigated for the presence of IBMF. Similarly, all adolescents with suspected MDS should be tested for underlying IBMF. All patients with IBMF should undergo serial assessment of peripheral blood and bone marrow including cytogenetics and NGS. Progressive cytopenia, development of MDS and increased percentage of bone marrow blasts, abnormal karyotype (3q+, −7,/7q, complex karyotype), RUNX1, and biallelic TP53 mutations are factors that identify risk patients and support the early indication of HSCT in IBMF (1, 6).

## Data Availability

The original contributions presented in the study are included in the article/supplementary material. Further inquiries can be directed to the corresponding author.
